# Cytotoxicity and reversal effect of sertraline, fluoxetine, and citalopram on MRP1- and MRP7-mediated MDR

**DOI:** 10.3389/fphar.2023.1290255

**Published:** 2023-11-02

**Authors:** Yuval Bin Kanner, Qiu-Xu Teng, Assaf Ganoth, Dan Peer, Jing-Quan Wang, Zhe-Sheng Chen, Yossi Tsfadia

**Affiliations:** ^1^ George S. Wise Faculty of Life Sciences, The School of Neurobiology, Biochemistry and Biophysics, Tel Aviv University, Tel Aviv, Israel; ^2^ Department of Pharmaceutical Sciences, College of Pharmacy and Health Sciences, St. John’s University, New York, NY, United States; ^3^ Department of Physical Therapy, Sackler Faculty of Medicine, School of Health Professions, Tel Aviv University, Tel Aviv, Israel; ^4^ Reichman University, Herzliya, Israel; ^5^ Laboratory of Precision NanoMedicine, George S. Wise Faculty of Life Sciences, Shmunis School for Biomedicine and Cancer Research, Tel Aviv University, Tel Aviv, Israel; ^6^ Center for Nanoscience and Nanotechnology, Tel Aviv University, Tel Aviv, Israel; ^7^ Department of Materials Sciences and Engineering, Iby and Aladar Fleischman Faculty of Engineering, Tel Aviv University, Tel Aviv, Israel; ^8^ Cancer Biology Research Center, Tel Aviv University, Tel Aviv, Israel

**Keywords:** cancer, drug resistance, SSRI family, ABC transporters, ABCC1/MRP1, ABCC10/MRP7

## Abstract

Cancer is one of the leading causes of death worldwide, and the development of resistance to chemotherapy drugs is a major challenge in treating malignancies. In recent years, researchers have focused on understanding the mechanisms of multidrug resistance (MDR) in cancer cells and have identified the overexpression of ATP-binding cassette (ABC) transporters, including ABCC1/MRP1 and ABCC10/MRP7, as a key factor in the development of MDR. In this study, we aimed to investigate whether three drugs (sertraline, fluoxetine, and citalopram) from the selective serotonin reuptake inhibitor (SSRI) family, commonly used as antidepressants, could be repurposed as inhibitors of MRP1 and MRP7 transporters and reverse MDR in cancer cells. Using a combination of *in silico* predictions and *in vitro* validations, we analyzed the interaction of MRP1 and MRP7 with the drugs and evaluated their ability to hinder cell resistance. We used computational tools to identify and analyze the binding site of these three molecules and determine their binding energy. Subsequently, we conducted experimental assays to assess cell viability when treated with various standard chemotherapies, both with and without the presence of SSRI inhibitors. Our results show that all three SSRI drugs exhibited inhibitory/reversal effects in the presence of chemotherapies on both MRP1-overexpressed cells and MRP7-overexpressed cells, suggesting that these medications have the potential to be repurposed to target MDR in cancer cells. These findings may open the door to using FDA-approved medications in combination therapy protocols to treat highly resistant malignancies and improve the efficacy of chemotherapy treatment. Our research highlights the importance of investigating and repurposing existing drugs to overcome MDR in cancer treatment.

## 1 Introduction

Cancer is a global health concern, with an estimated 19.3 million new cases and almost 10 million deaths reported in 2020 (Sung et al., 2021). Chemotherapy is a common treatment for cancer, but its success depends on reaching an effective anticancer drug concentration inside the cancer cells. Unfortunately, cancers are capable to develop resistance to a variety of chemotherapeutic agents, severely reducing the efficiency of the treatment. Research in recent decades has been focused on understanding the phenomenon of multidrug resistance (MDR) in cancer cells (Nooter and Stoter, 1996; Wu et al., 2011). One of the main causes of MDR is the overexpression of transporters on the membrane of cancer cells that actively export the anti-cancer drugs out of the tumor cells, thereby increasing their survival rates (Leslie et al., 2001; Austin Doyle and Ross, 2003; Thomas and Coley, 2003; Leslie et al., 2005; Munoz et al., 2007). Those pumps belong to the ATP-Binding Cassette (ABC) transporters superfamily, which its members utilize binding and hydrolysis of ATP to translocate substrates across membranes (Tiwari et al., 2011; Wilkens, 2015).

The human genome organization divides the currently 49 known human ABC transporters into seven subfamilies, from ABCA to ABCG. The subfamily ABCC contains 13 members, nine of which are referred to as Multidrug Resistance Proteins (MRPs) (Dean and Allikmets, 2001). Based on their structures, the MRPs are classified as short MRPs (MRP4, MRP5, MRP8, and MRP9) and long MRPs (MRP1, MRP2, MRP3, MRP6, and MRP7). The structure of MRPs is composed of two transmembrane domains (TMDs) and two nucleotide-binding domains (NBDs) that are responsible for binding and hydrolyzing ATP. The NBDs are located on the cytoplasmic side of the membrane, while the TMDs span the lipid bilayer and form the substrate-binding site. The NBDs and the TMDs are connected by a linker region (Wilkens, 2015). The long MRPs are characterized by an additional fifth domain, TMD0, located at the N-terminus of the transporters (Johnson and Chen, 2018). Two of the MRPs, ABCC1/MRP1, and ABCC10/MRP7 have been found to be highly expressed in a variety of tissues, such as the liver, kidney, and brain (Kruh and Belinsky, 2003). They have also been shown to transport a wide range of substrates, including drugs, toxins, and other small molecules. They actively extrude glucuronide conjugates such as estradiol-17-beta-o-glucuronide, glutathione conjugates, such as leukotriene C4 (LTC4), and other xenobiotics (Leier et al., 1994; Stride et al., 1997; Sjölinder et al., 1999; Chen et al., 2003; Hopper-Borge et al., 2004). Both mediate MDR in cancer cells by preventing the intracellular accumulation of anticancer drugs (Stride et al., 1997; Hopper-Borge et al., 2004; Malofeeva et al., 2012).

Inhibiting the function of ABC transporters using small molecules is considered a potential strategy for reversing MDR (Palmeira et al., 2012; Peña-Solórzano et al., 2017; Stefan and Wiese, 2019). Chemosensitizers are drugs that are designed to make cancer cells more susceptible to chemotherapy. By using chemosensitizers in combination with chemotherapy, the efficacy of treatment can be improved, and resistance can be overcome (Krishna and Mayer, 2000). Previous studies have demonstrated that drugs that are not specifically designed for cancer treatment can have anti-cancer effects and thus can be repurposed: Metformin, a drug used to treat type 2 diabetes, has been shown to have anti-cancer properties and has been found to increase the sensitivity of cancer cells to chemotherapy in preclinical and clinical studies (Aljofan and Riethmacher, 2019; Cheng et al., 2020; Urpilainen et al., 2020); Sildenafil, a phosphodiesterase-5 inhibitor used to treat erectile dysfunction, can sensitize cancer cells to chemotherapy and radiation therapy, making them more vulnerable to treatment (Das et al., 2010; Di et al., 2010; Pantziarka et al., 2018; Cruz-Burgos et al., 2021); Statins, drugs used to lower cholesterol levels, have also been found to have potential as chemosensitizers, by increasing the sensitivity of cancer cells to chemotherapy, leading to improved treatment outcomes (Oku et al., 2015; Mallappa et al., 2019).

Selective Serotonin Reuptake inhibitors (SSRIs) are a class of medications commonly used to treat depression, anxiety, and other mental health conditions. These medications work by increasing the levels of the neurotransmitter serotonin in the brain, which helps to reduce symptoms of mental health conditions. They are generally considered to have fewer side effects and be less dangerous than older-generation antidepressants, such as tricyclic antidepressants, because they specifically target the serotonin transporter, rather than affecting a wide range of neurotransmitters (Wang et al., 2018). It was previously shown that drugs from the SSRI family exhibit anti-cancer properties (Nordenberg et al., 1999; Serafeim et al., 2003; Argov et al., 2009; Kast et al., 2013; Zhang et al., 2022). Moreover, it was suggested that SSRI members can be repositioned for cancer treatment (Peer and Margalit, 2006; Drinberg et al., 2014; Baú-Carneiro et al., 2022). For example, fluoxetine (Prozac) was found to be unique among other repurposed chemosensitizers, in that it can effectively target MDR cells at low, safe doses that are well below the levels considered safe for humans. It was proposed this could potentially merit a separate classification for fluoxetine, and maybe other SSRIs, as a fourth-generation chemosensitizer (Peer and Margalit, 2006). Based on these findings, we sought to investigate whether members of the SSRI family, sertraline, fluoxetine, and citalopram, could modulate resistance in cells overexpressing MRP1 and MRP7 transporters.

In this study, we use a multi-disciplinary approach to investigate the protein-ligand interaction of MRP1 and MRP7 with these three SSRI medications and evaluate their ability to counter cell resistance. We used *in silico* tools to predict the binding site of each molecule and performed energy calculations to evaluate the complexes’ stability. Then, using *in vitro* assays we were able to examine cancer cell viability using each of the molecules combined with different commonly used chemotherapies. Our results demonstrate that all three SSRI drugs had inhibitory/reversal effects on both MRP1-overexpressed cells and MRP7-overexpressed cells. Hence, our findings suggest that these FDA-approved medications may have potential therapeutic benefits when used in combination therapy protocols to treat highly resistant malignancies. Although there are several MRP1 inhibitors that have been investigated in preclinical and clinical studies, there are currently no MRP1 inhibitors that are approved for clinical use as chemosensitizers (Wang et al., 2021a). Given the high costs and the low success rates associated with traditional drug development schemes, repurposing existing drugs for use as chemosensitizers is a cost-effective and efficient approach to identifying new chemosensitizers. The study opens new possibilities to repurpose these drugs to address this unmet medical need.

## 2 Materials and methods

### 2.1 Protein structures and evaluation

The hMRP1 (https://alphafold.ebi.ac.uk/entry/P33527) and hMRP7 (https://alphafold.ebi.ac.uk/entry/Q5T3U5) structures were predicted using AlphaFold (Jumper et al., 2021). Despite the availability of cryo-EM structures of bovine MRP1 in complex with and without the ligand LTC4 (Johnson and Chen, 2017), no high-resolution human MRP1 structure has been determined to date. These structures also contain missing residues, such as an 88-residue gap between residues 868 and 955. The AlphaFold prediction for MRP1 closely resembles the bovine MRP1 structure in its bound state, with an RMSD value of 1.707 Å. As no high-resolution human MRP7 structure is currently available, we also utilized the AlphaFold algorithm to model this protein.

Evaluation of the AlphaFold predicted model protein structures was performed using pLDDT (predicted Local Distance Difference Test) (Jumper et al., 2021), ProSA (Sippl, 1993), ERRAT (Colovos and Yeates, 1993), Procheck 3.5.4 (Laskowski et al., 1993), and assessment tools (clashscore and MolProbity (Williams et al., 2018)) integrated within the SWISS-MODEL workspace (Waterhouse et al., 2018).

### 2.2 Protein structure network elastic network model (PSN-ENM)

Protein Structure Network (PSN) and Elastic Network Model (ENM) are computational biology techniques used to study the dynamic behavior of proteins (Raimondi et al., 2013). In the PSN model, the protein structure is depicted as a network of nodes and edges. The nodes correspond to amino acid residues, while the edges represent their interactions. The ENM is a coarse-grained model of the protein structure that simplifies the interactions between atoms. It enables the calculation of the normal modes of protein motion, which offer insight into the protein’s dynamics and flexibility. We used the WebPSN (Seeber et al., 2015; Felline et al., 2020) for calculating the interactions between the residues in the protein and mapping these interactions onto a network (Seeber et al., 2015; Felline et al., 2020). The resulting network representation of the protein structure was applied to compare MRP1 and MRP7 and identify critical residues and regions at the TMDs.

### 2.3 Normal mode analysis (NMA)

NMA was performed to characterize the inherent flexibility of MRP1 and MRP7 using DynaMut (https://biosig.lab.uq.edu.au/dynamut/). This extracts the atomic displacement of the Cα atoms and their relative motion amplitude in order to account for their intrinsic motions (Rodrigues et al., 2018). Statistical comparison between the obtained flexibility measures was calculated using Student’s t-test.

### 2.4 Protein preparation and grid generation for docking

Bond orders were assigned, hydrogen atoms were added, water molecules were removed beyond 5 Å of the molecule, and energy minimization was applied using the OPLS4 force field. A receptor grid for all the ligands was generated using the Glide application (Schrodinger Release, 2021a) of Maestro (Schrodinger Release, 2021b) within Schrödinger suite (Schrodinger Release, 2021c) by specifying the highest score binding (active) site residues identified by the “SiteMap” tool (Schrodinger Release, 2021d).

### 2.5 Ligand preparation for docking

Three FDA-approved drugs from the SSRI family (Sertraline, Fluoxetine, and Citalopram) and two known chemotherapeutic drugs (Doxorubicin and Vincristine) were selected for molecular docking. PubChem database (https://pubchem.ncbi.nlm.nih.gov) was used to extract the chemical structure of the selected molecules. Using the LigPrep module (Schrodinger Release, 2021e) from the Maestro (Schrodinger Release, 2021f), the ligands’ structures were optimized using the OPLS4 force field, with respect to energy, chirality, and ionization state. Once all the ligands were optimized, they were further considered for docking studies.

The pKa values of Sertraline, Fluoxetine, and Citalopram, which were determined using Chemaxon (https://chemaxon.com) lie within the range of 9–10. At a physiological pH (7.35–7.45), it is likely that these three molecules would exist predominantly in their protonated (acidic) form and would have a positive charge.

### 2.6 Molecular docking

Flexible docking was performed on a defined receptor grid using the extra precision mode (XP) in the Glide application (Schrodinger Release, 2021a) of Maestro (Schrodinger Release, 2021b). The glide energy was used to rank the various docking poses and identify the ones that are likely to represent the most favorable binding poses in the protein-ligand complexes. The structure output format was set to a pose viewer file to view the output of the resulting docking studies from a pose viewer.

### 2.7 Halogen bonds

To analyze the existence of halogen bonds between Sertraline, Fluoxetine, and Citalopram to MRP1 and MRP7, we used Protein–Ligand Interaction Profiler (PLIP) (https://plip-tool.biotec.tu-dresden.de/plip-web/plip/index). PLIP is a Python-based open-source software that provides a detailed report on the intermolecular interactions between a protein and its ligand. It identifies different types of interactions such as hydrogen bonds, salt bridges, pi-stacking, hydrophobic contacts and halogen bonds (Salentin et al., 2015; Adasme et al., 2021).

### 2.8 Molecular mechanics-generalized born surface area (MM-GBSA) calculations

MM-GBSA calculations were performed using two different tools: the Prime module (Schrodinger Release, 2021f) in the Schrödinger Maestro (Schrodinger Release, 2021e) and the fastDRH (http://cadd.zju.edu.cn/fastdrh/overview) (Wang et al., 2022). These tools were used to compute the binding energies using the MM-GBSA method, which involves two equations:

$$∆G_{\text{bind}}=G_{\text{Complex}}\;–\;\left(G_{\text{Receptor}}+G_{\text{Ligand}}\right)$$ (1)

The MM-GBSA free energy of binding (∆G_bind_) is calculated as the difference between the free energy of the complex (G_Complex_), and the sum of the free energies of the receptor (G_Receptor_), and the ligand (G_Ligand_), in solution (Genheden and Ryde, 2015).

$$G_{\text{Molecule}}=∆E_{\text{MM}}+∆G_{\text{GB}}+∆G_{\text{SA}}\;–\;T∆S$$ (2)

Equation 2 represents the free energy of the molecule. It takes into account the change in internal energy of the molecule (∆E_MM_) calculated using molecular mechanics, the change in solvation energy of the molecule (∆G_GB_) calculated using the generalized Born surface area method, the change in surface area of the molecule (∆G_SA_), and the change in entropy of the molecule (T∆S) (Genheden and Ryde, 2015).

### 2.9 Materials

Sertraline, fluoxetine, and citalopram were purchased from TCI America (Portland, OR, USA). Doxorubicin was purchased from LC laboratories (Woburn, MA, USA). Paclitaxel, vincristine, and cisplatin were purchased from Alfa Aesar (Tewksbury, MA, USA). Dimethyl sulfoxide (DMSO) and 3-(4,5-dimethylthiazole-2-yl)-2,5-biphenyltetrazolium bromide (MTT) were purchased from Sigma Chemical Co. (St. Louis, MO, USA). Phosphate buffer saline (PBS), Dulbecco’s modified Eagle’s medium (DMEM), RPMI-1640 with L-glutamine medium (RPMI-1640), fetal bovine serum (FBS), penicillin/streptomycin and trypsin-EDTA 0.25% were purchased from Hyclone (Waltham, MA, USA).

### 2.10 Cell lines and cell culture

This study used the human epidermoid carcinoma cell line KB-3-1 as the drug-sensitive cell line, and its MRP1-overexpressing cell line KB/CV60 (Supplementary Figure S1A), which was maintained in the medium with 1 μg/mL of cepharanthine and 60 ng/mL of vincristine (Aoki et al., 2001); and the MRP7-overexpressing SKOV3/MRP7 cell line (Supplementary Figure S1B) by transfecting recombinant pcDNA3.1/MRP7 plasmids and the parental human ovarian adenocarcinoma cell line SKOV3 (Wang et al., 2021b) were also used. All cell lines were grown as adherent monolayers in an essential medium supplemented with 10% FBS and 1% penicillin/streptomycin in a humid atmosphere incubator at 37 °C with 5% CO_2_. KB-3-1 and KB/CV60 were cultured in DMEM, and SKOV3 and SKOV3/MPR7 were cultured in RPMI-1640.

### 2.11 Cytotoxicity determination by MTT assay

The cytotoxicity was analyzed using a slightly modified MTT assay as previously described (Wang et al., 2021c). Cells were collected and resuspended at a final concentration of 5 × 10^3^ cells/well for all four kinds of cell lines. Paclitaxel, doxorubicin, and vincristine, known as MRP7 or MRP1 substrate drugs, were used as positive controls for different cell lines, and cisplatin as non-substrate for both MRP7 and MRP1 was used as the negative control. The absorbance was determined at 570 nm by the accuSkan GO UV/Vis Microplate Spectrophotometer (Fisher Sci., Fair Lawn, NJ). The IC_50_ values were calculated from the survival curves.

## 3 Results

**FIGURE 1:**
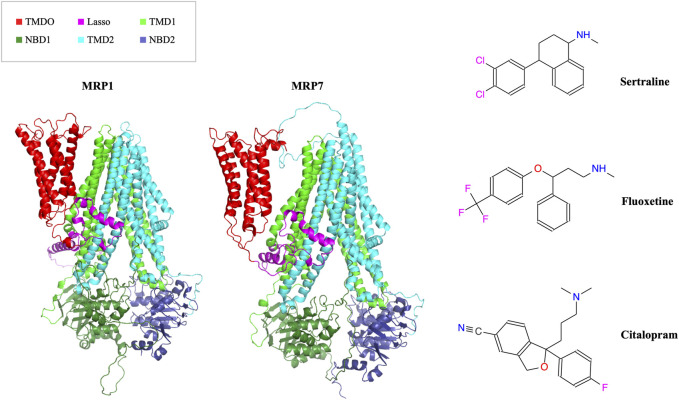
In the left panel, the AlphaFold model structures of MRP1 and MRP7 are presented in a cartoon representation viewed from within the membrane plane. Protein’s domains: TMD0, Lasso motif, TMD1, NBD1, TMD2, and NBD2 are colored in red, pink, light green, dark green, cyan, and blue, respectively. In the right panel, the molecular structures of three clinically used SSRI medications (sertraline, fluoxetine, and citalopram) are shown.

In this study, we aimed to determine if three drugs from the SSRI family (sertraline, fluoxetine, and citalopram), commonly used as antidepressants (Figure 1, right panel), can be repurposed as inhibitors of MRP1 and MRP7 transporters (Figure 1, left panel) to overcome MDR in cancer cells.

### 3.1 Quality assessments and flexibility of the human MRP1 and MRP7 AlphaFold’s structures

The AI-generated models of the human MRP1 and MRP7 were evaluated by validation means for their quality. AlphaFold generates a confidence score for each residue, known as pLDDT, which ranges from 0 to 100. The higher the pLDDT score, the more confidence the model has in its prediction. The pLDDT scores for the entire proteins’ sequences are 83.01 ± 16.10 and 81.66 ± 16.54 for MRP1 and MRP7, respectively. When considering only the core of the protein (TMD1+2 and NBD1+2), the pLDDT score increases to 87.63 ± 7.54 and 85.63 ± 13.42 for MRP1 and MRP7, respectively. The modeling accuracy is highly supported by the elevated confidence levels, which in turn significantly enhance the likelihood of correctly localizing binding sites (Jumper et al., 2021). ProSA analysis of MRP1 and MRP7 structures gave Z-scores of −13.46 and −15.17, respectively, indicating their structures lie in the range compared to similar-sized structures in the PDB database. ERRAT analysis revealed an overall quality of 97.16% and 95.99% for the MRP1 and MRP7 structures, respectively, when any value above 95% indicates a high confidence level. All bond lengths, backbone, and rotamer angles were in high agreement with standard values. For both proteins, no major stereochemical clashes or bad contacts for main-chain or side-chain parameters were detected. The clashscore and the MolProbity score were very low (1.13 and 1.31 respectively for MRP1, and 0.48 and 0.98 for MRP7), indicating high-quality models. Ramachandran plot for MRP1 showed that 99.7% of the residues lie in allowed regions, from which 92.8% in the most favored regions, whereas for the MRP7 99.8% of the residues lie in allowed regions, from which 92.6% in the most favored regions. Overall, according to our quality assessment analyses the AlphaFold-generated models of both proteins are considered reliable structures.

NMA was used to capture the intrinsic flexibility of MRP1 and MRP7. Our results suggest that MRP1 is much more flexible than MRP7 (*p*-value is <.00001), and it appears to possess a greater degree of conformational adaptability. This is evidenced by having dynamic regions that facilitate its capacity to interact with and transport a diverse range of substrates. Conversely, MRP7 exhibits a comparatively rigid architecture, featuring fewer flexible regions, which may limit its substrate selectivity and transport efficacy.

### 3.2 Comparison of the human MRP1 and MRP7 using the PSN-ENM approach

In this study, we employed the PSN-ENM (Protein Structure Network - Elastic Network Model) approach to compare the human MRP1 and MRP7 as receptors, with the aim of identifying similarities and differences between these two proteins. We conducted two types of analysis: hubs analysis and meta-path analysis, both of which yielded significant insights.

**FIGURE 2:**
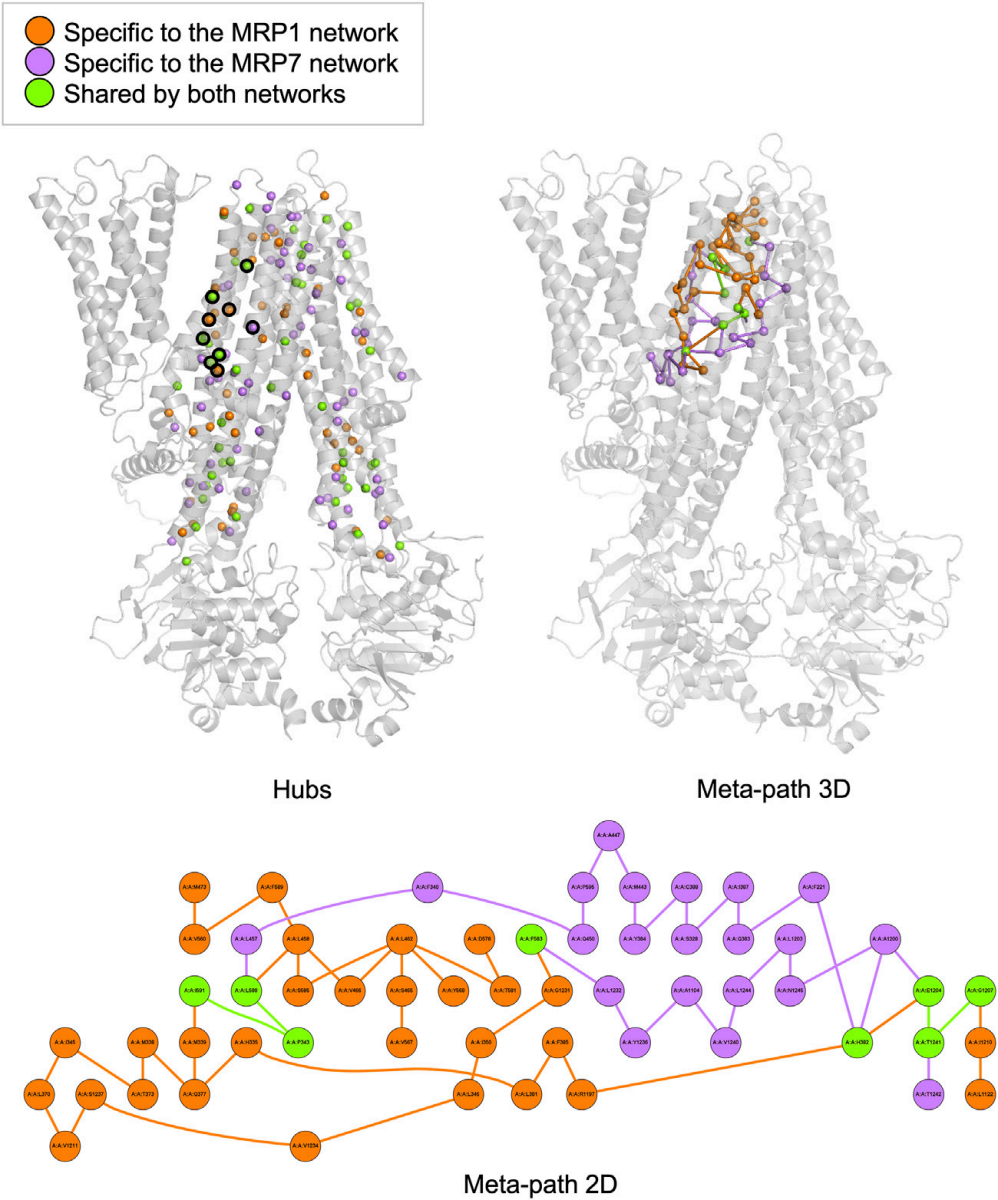
Hubs and meta-path analysis projection at the MRP1 structure (as a representative for both MRP1 and MRP7 structures) colored by the following color code: orange - specific for MRP1, purple - specific for MRP7 and green - mutual for both proteins. A 3D representation of MRP1 shows the hubs located in TMD1 and TMD2 as spheres (left panel). Hubs discussed in the text are surrounded by a black frame. A 3D representation of MRP1 shows the meta-path as a series of lines connecting the different nodes (right panel). A 2D representation of the meta-path presents a more detailed view of the interactions between residues along communication pathways in MRP1 (lower panel).

Hubs analysis refers to the identification of specific residues in a protein that have a relatively large number of connections or interactions with other residues in the same protein. These interactions can be in the form of hydrogen bonds, salt bridges, van der Waals forces, and hydrophobic interactions. We found that MRP1 and MRP7 have distinct sets of hubs, with 234 and 251 total hubs, respectively, and 111 shared hubs between the two proteins. Our analysis identified 46 shared hubs between MRP1 and MRP7 in the transmembrane domains TMD1 and TMD2, which are likely to be the “hot spots” that interact with potential ligands (Figure 2, left panel). For instance, we found that certain residues in MRP1 (K332, M338, P343, H382, and Y384) correspond to specific residues in MRP7 (K292, L298, P303, H342, and Y344). On the other hand, unique hubs for each protein are likely to be crucial in determining their binding specificity. For example, we found that H335, F385, and Q450 are unique hubs for MRP1, while Q1156 is a unique hub for MRP7.

Meta-path analysis refers to the identification of sequences of residues within a protein that are connected by specific interactions. Our meta-path analysis revealed that MRP1 and MRP7 had different dominant meta-paths. Specifically, we found that MRP1 had more diverse and complex meta-paths than MRP7, with 38 links compared to 28 links in MRP7. Furthermore, only four links and eight nodes were shared by both meta-paths, indicating differences in their inner pathways of communication between residues. The common links for the two proteins are I591-P343, L588-P343, G1207-T1241, and E1204-T1241 in MRP1 and M546-P303, L543-P303, G1159-T1195, and Q1156-T1195 in MRP7, respectively. The mutual nodes within the meta-paths are P343, H382, F583, L588, I591, E1204, G1207, and T1241 in MRP1 and P303, N342, F538, L543, M546, Q1156, and T1195 in MRP7, respectively.

The classification of unique hubs and meta-paths through our analysis can aid in the identification and selection of key residues for drug design targeting MRP1 and MRP7 proteins.

### 3.3 Identifying binding pockets of sertraline, fluoxetine, and citalopram with MRP1 and MRP7 through molecular docking

**FIGURE 3:**
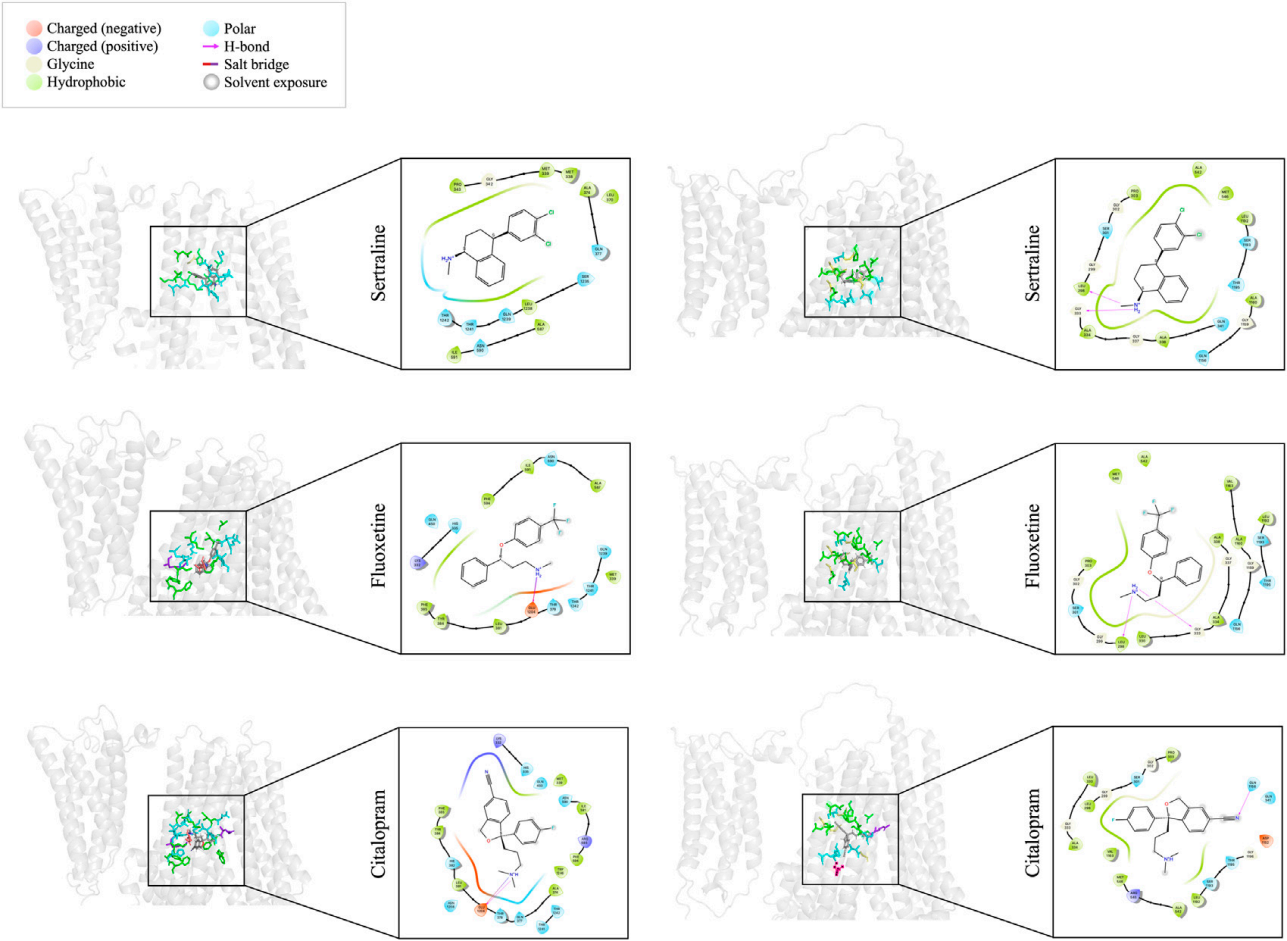
Docking interactions of sertraline, fluoxetine, and citalopram with MRP1 (left panel) and MRP7 (right panel). On the left side of each panel, a 3D cartoon representation of the extracellular part of the protein (in light gray), bound to the different drugs (sticks representation in dark gray) is presented. Residues involved in the binding of each drug are colored as described in the color legend. On the right side of each panel, a 2D representation of the different interactions of the specific drug in the binding site, including residues’ names and positions, are colored based on the color legend.

We utilized molecular docking to analyze the binding of sertraline, fluoxetine, and citalopram with MRP1 and MRP7, resulting in a total of six protein-ligand complexes. Docking was performed on a defined receptor grid using the extra precision mode (XP) in the Glide application as described in the Methods section. The binding site of MRP1 to the three inhibitors is mainly composed of hydrophobic and polar residues (Figure 3, left panel). The hydrophobic residues involve in sertraline binding are P343, M338, M339, L370, A374, A587, I591, and L1238. Additionally, there are polar regions composed of the following residues: Q377, N590, S1235, Q1239, T1241, and T1242. G342 is also part of the sertraline binding site. The binding site of fluoxetine (Figure 3, left panel, middle row) is also composed of hydrophobic and polar residues as well as charged ones. The hydrophobic residues involve in fluoxetine binding are M339, L381, Y384, F385, A587, I591, and F594. In addition, there are polar regions composed of the following residues: H335, T378, Q450, N590, Q1239, T1241, and T1242. The positively charged K332 and the negatively charged E1204 are also stabilizing fluoxetine in the binding site. Similarly, the binding site of citalopram (Figure 3, left panel, bottom row) is composed of hydrophobic, polar, and charged residues. The hydrophobic residues involve in citalopram binding are M339, A374, L381, Y384, F385, I591, F594, and W1246. The polar residues involve in citalopram binding are HIS335, Q450, N590, Q377, T378, H382, N1208, T1241, and T1242. The positively charged K332 and R593 as well as the negatively charged E1204 also play a role in the fluoxetine binding.

The binding site of MRP7 to sertraline shares similarities with MRP1, as it is mainly composed of hydrophobic and polar residues. However, the binding site of MRP7 includes five glycine residues, whereas only one glycine residue was involved in the interactions of MRP1 (Figure 3, right panel, top row). The hydrophobic residues involve in sertraline binding are L298, P303, A334, A338, A542, M546, A1160, and L1192. The polar regions are composed of the following residues: S301, Q341, Q1156, S1193, and T1195. The glycine residues that are involved in the binding are in positions 299, 302, 333, 337, and 1159. Similarly, the binding site of fluoxetine to MRP7 (Figure 3, right panel, middle row) is composed of hydrophobic residues, polar residues, and glycine residues. The hydrophobic residues involve in fluoxetine binding are L298, P303, L330, A334, A338, A542, M546, A1160, V1163, and L1192. The polar regions are composed of the following residues: S301, Q1156, S1193, and T1195. The glycine residues are the same as in the sertraline binding site. The binding site of citalopram to MRP7 (Figure 3, right panel, bottom row) is composed of hydrophobic, polar, charged, and glycine residues. The hydrophobic residues involve in citalopram binding are the same as for fluoxetine, except for A338 and A1160. The polar residues involved in citalopram binding are the same as for sertraline. The glycine residues that are involved in the binding are in positions 299, 302, 333, and 1196. The positively charged R545 and the negatively charged D1152 are also stabilizing the citalopram in the binding site.

### 3.4 Comparison of the interacting residues in the binding sites of MRP1 and MRP7

The amino acids of MRP1 and MRP7 that are involved in the binding of the drugs sertraline, fluoxetine, and citalopram are summarized in Table 1. From the proteins’ perspective: MRP1 and MRP7 share three residues that interact with all the drugs: M339, I591, and T1241 in MRP1 correspond to G299, M546, and T1195 in MRP7 (Table 1, dark gray). For MRP1, residues N590 and T1242 are involved in binding all three ligands but not for MRP7 (Table 1, light gray). For MRP7 solely, the residues that are involved in the binding of all three ligands are L298, S301, G302, P303, A334, A542, Q1156, L1192, and S1193 (Table 1, light gray). From the drugs’ perspective, there are 10 mutual residues in MRP1 and MRP7 that bind sertraline: M338/L298, M339/G299, G342/G302, P343/P303, A374/A334, Q377/G337, A587/A542, I591/M546, L1238/L1192, Q1239/S1193, and T1241/T1195 for MRP1/MRP7 respectively; seven mutual residues in MRP1 and MRP7 that bind fluoxetine: M339/G299, R378/A338, A587/A542, I591/M546, E1204/Q1156, Q1239/S1193, and T1241/T1195; eight mutual residues in MRP1 and MRP7 that bind citalopram: M339/G299, A374/A334, L381/Q341, N590/R545, I591/M546, E1204/Q1156, T1241/T1195, and T1242/G1196.

**TABLE 1:** The interacting residues involved in binding the three inhibitors, sertraline, fluoxetine, and citalopram, to the proteins MRP1 and MRP7. Each row presents a different amino acid and its position, and each column presents a different drug. The presence of an amino acid is indicated by a letter and a number, and its absence is indicated by a hyphen. The MRP1 and MRP7 were aligned using PyMOL, and the table uses color coding to highlight residues found to bind the inhibitors. Residues that bind the three inhibitors in the same protein are colored in light gray, and residues that bind the three inhibitors in both proteins are colored in dark gray. Residues binding the same drug for both proteins are highlighted in bold.

MRP1			MRP7		
Sertraline	Fluoxetine	Citalopram	Sertraline	Fluoxetine	Citalopram
-	K332	K332	-	-	-
-	H335	H335	-	-	-
**M338**	-	-	**L298**	L298	L298
**M339**	**M339**	**M339**	**G299**	**G299**	**G299**
-	-	-	S301	S301	S301
**G342**	-	-	**G302**	G302	G302
**P343**	-	-	**P303**	P303	P303
L370	-	-	-	L330	L330
-	-	-	G333	-	G333
**A374**	-	**A374**	**A334**	A334	**A334**
**Q377**	-	Q377	**G337**	G337	-
-	**T378**	T378	A338	**A338**	-
-	L381	**L381**	Q341	-	**Q341**
-	-	H382	-	-	-
-	Y384	Y384	-	-	-
-	F385	F385	-	-	-
-	Q450	Q450	-	-	-
**A587**	**A587**	-	**A542**	**A542**	A542
N590	N590	**N590**	-	-	**R545**
**I591**	**I591**	**I591**	**M546**	**M546**	**M546**
-	-	R593	-	-	-
-	F594	F594	-	-	-
-	-	-	-	-	D1152
-	**E1204**	**E1204**	Q1156	**Q1156**	**Q1156**
-	-	-	G1159	G1159	-
-	-	N1208	A1160	A1160	-
-	-	-	-	V1163	-
S1235	-	-	-	-	-
**L1238**	-	-	**L1192**	L1192	L1192
**Q1239**	**Q1239**	-	**S1193**	**S1193**	S1193
**T1241**	**T1241**	**T1241**	**T1195**	**T1195**	**T1195**
T1242	T1242	**T1242**	-	-	**G1196**
-	-	W1246	-	-	-

MRP1 and MRP7 share a significant amount of corresponding residues, involved in the binding of sertraline, fluoxetine, and citalopram. This suggests that both MRP1 and MRP7 may bind these drugs in similar ways and may have similar mechanisms of drug resistance. The amino acids that exclusively interact with the drugs only in one protein can provide crucial insights into the specific interactions that facilitate the binding of these drugs to MRP1, in contrast to MRP7.

### 3.5 Halogen-mediated interactions

**FIGURE 4:**
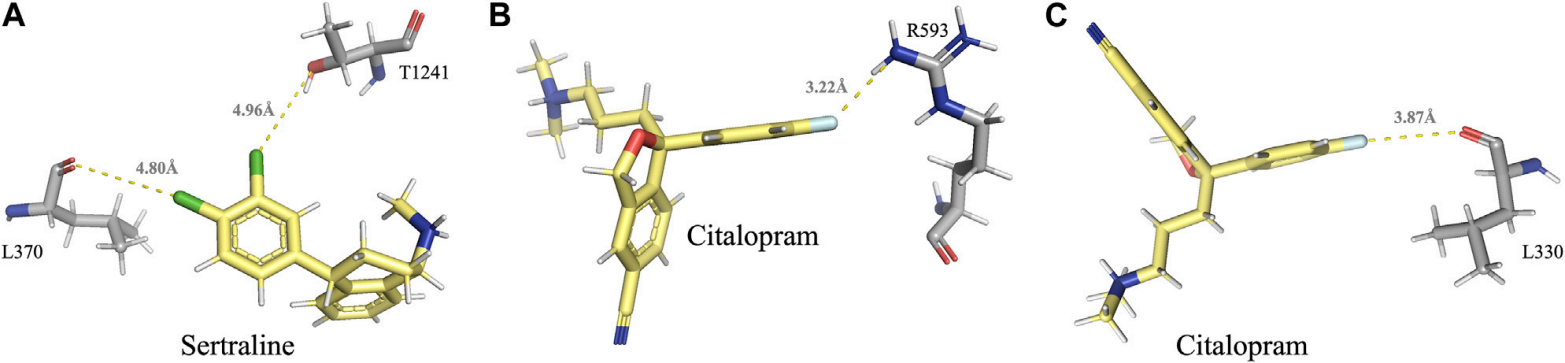
Visual representation of the halogen bonds formed by sertraline and citalopram with MRP1 and MRP7, as elaborated in table 2. **(A)** Halogen bonds formed between the chlorine atoms of sertraline and the backbone carbonyl oxygen atom of L370 and the side-chain hydroxyl oxygen atom of T1241. **(B)** A halogen bond formed between the fluorine atom of citalopram and the guanidinium group of R593. **(C)** A halogen bond formed between the fluorine atom of citalopram and the backbone carbonyl oxygen of L330. Residues of MRP1 and MRP7 are colored in gray, and the drugs are colored in yellow.

A halogen bond is a type of intermolecular interaction that results from the electrostatic interaction between a halogen atom and a negatively charged species, such as a lone pair of electrons or a negative ion. XB donors can include iodine, bromine, chlorine, and, in certain cases, fluorine. Although the fluorine atom is not a very effective XB donor, it can exhibit a positive σ-hole when bound to another fluorine atom or attached to O, N, C, or other atoms that possess strong electron-withdrawing substituents (Cavallo et al., 2016). Halogen bonds share similarities with hydrogen bonds in terms of strength and directionality; however, they possess a longer range (in some cases up to 6.0 Å) and exhibit greater directionality. In protein-ligand complexes, halogen bonding interactions can arise between a halogen-containing ligand and any available Lewis base within the protein’s binding site (Wilcken et al., 2013). Sertraline, fluoxetine, and citalopram share a common feature in their chemical structure - the presence of halogen groups. Specifically, sertraline has a dichlorophenyl group, fluoxetine has a trifluoromethyl group, and citalopram has a fluorophenyl group. Our analyses have shown that these groups play a significant role in the interactions with the MRPs. All halogen bonds were analyzed using Protein–Ligand Interaction Profiler (PLIP) and subsequently validated for correct orientation and bond length using PyMOL. Sertraline’s chlorine atoms interact with the backbone carbonyl oxygen atom of L370 (d_F↔O_ = 4.80 Å) and with the side-chain hydroxyl oxygen atom of T1241 (d_F↔O_ = 4.96 Å) in MRP1. Citalopram’s fluorine atom forms a halogen bond with the guanidinium group of R593 in MRP1 (d_F↔N_ = 3.22 Å) and with the backbone carbonyl oxygen of L330 in MRP7 (dF↔O = 3.87 Å) (Table 2; Figure 4).

**TABLE 2:** Halogen bonds formed by sertraline and citalopram with MRP1 and MRP7. Each row in the table includes specific details about the protein residues that are involved in the binding, the atoms of the ligand that participate in the interaction, and the geometry of the bond. All atom and residue numbering are in accordance with the numbering in the corresponding PDB file given as input (post-docking complex).

	Residue	AA	Distance (Å)	Donor angle	Acceptor angle	Donor atom	Acceptor atom
MRP1-SER	370	LEU	4.80	161.40	143.74	2 [Cl]	3065 [O]
	1241	THR	4.96	149.65	92.00	1 [Cl]	9826 [Oγ]
MRP1-CIT	593	ARG	3.22	140.51	114.03	1 [F]	4827 [Nε]
MRP7-CIT	330	LEU	3.87	151.46	145.53	1 [F]	2547 [O]

### 3.6 Energy calculations of the six complexes

To evaluate the binding affinity of these complexes, we used three different methods for energy calculations: Glide energy, Prime MM-GBSA calculations, and fastDRH MM-GBSA calculations (Table 3). The binding free energy value serves as an indicator of the binding strength between the ligand and protein, wherein a lower value corresponds to a more favorable docking orientation and a stronger binding interaction. A negative value signifies that the free energy of the protein-ligand complex is lower than that of the individual components when they are present in a solution, indicating greater stability and a higher probability of being observed in nature.

**TABLE 3:** The energy values (kcal/mol units) of the three SSRI drugs (sertraline, fluoxetine, and citalopram) bound to MRP1 and MRP7, as calculated by three different methods: Glide Energy, MM-GBSA using Prime, and MM-GBSA using fastDRH. A more favorable binding position is indicated by a lower energy value. The table presents the top-ranked pose/most favorable docking conformer for each protein-ligand complex.

	Glide energy		MM-GBSA (Prime)		MM-GBSA (fastDRH)	
**Protein**	MRP1	MRP7	MRP1	MRP7	MRP1	MRP7
**Drug**						
Sertraline	−33.516	−31.386	−40.35	−43.17	−31.22	−25.66
Fluoxetine	−30.592	−36.024	−33.64	−37.24	−22.60	−30.87
Citalopram	−35.401	−33.102	−42.70	−35.83	−28.21	−25.67

The Glide energy scores were analyzed to measure the quality of the docking pose, considering various factors such as the shape complementarity between the ligand and the protein binding site, the van der Waals interactions strength, and the solvation energy of the complex. All six complexes had comparable Glide energy scores ranging from −36.024 to −30.592 kcal/mol. The MRP1-sertraline and MRP1-citalopram complexes had better docking scores than the MRP7 complexes. However, the MRP7-fluoxetine complex had a better score than the MRP1 complex. Among the six complexes, the MRP7-fluoxetine complex achieved the best Glide energy score, whereas the MRP1-fluoxetine complex had the least favorable score.

The Prime MM-GBSA calculations were performed to validate the docking results further. All inhibitors had relatively similar binding free energy (∆G_Bind_) values ranging from −43.17 to −33.64 kcal/mol. The MRP7-sertraline and MRP7-fluoxetine complexes had lower free energy of binding values than the MRP1 complexes. However, the MRP1-citalopram complex had a lower free energy of binding value than the MRP7 complex. Among the complexes, the MRP7-sertraline complex had the most negative free energy of binding value (−43.17 kcal/mol), whereas the MRP1-fluoxetine complex had the least negative value (−33.64 kcal/mol).

In addition, we used the fastDRH software, an independent tool from the Schrödinger suite, to compute the binding energy score. The results showed that the MM-GBSA binding energy using fastDRH ranged from −31.22 to −22.6 kcal/mol, with a variation similar to that obtained by the Glide energy and the MM-GBSA using Prime. The MRP1-sertraline and MRP1-citalopram complexes exhibited lower free energy of binding values than the MRP7 complexes, but the MRP7-fluoxetine complex had a lower value than the MRP1 complex. The MRP1-sertraline complex displayed the most negative value (−31.22 kcal/mol), whereas the MRP1-fluoxetine complex had the least negative value (−22.60 kcal/mol). The slight difference in the values between the two methods for MM-GBSA calculations is algorithm dependent.

### 3.7 The effect of sertraline, fluoxetine, and citalopram on the efficacy of antineoplastic drugs in MRP1- and MRP7-Mediated MDR cell lines

**FIGURE 5:**
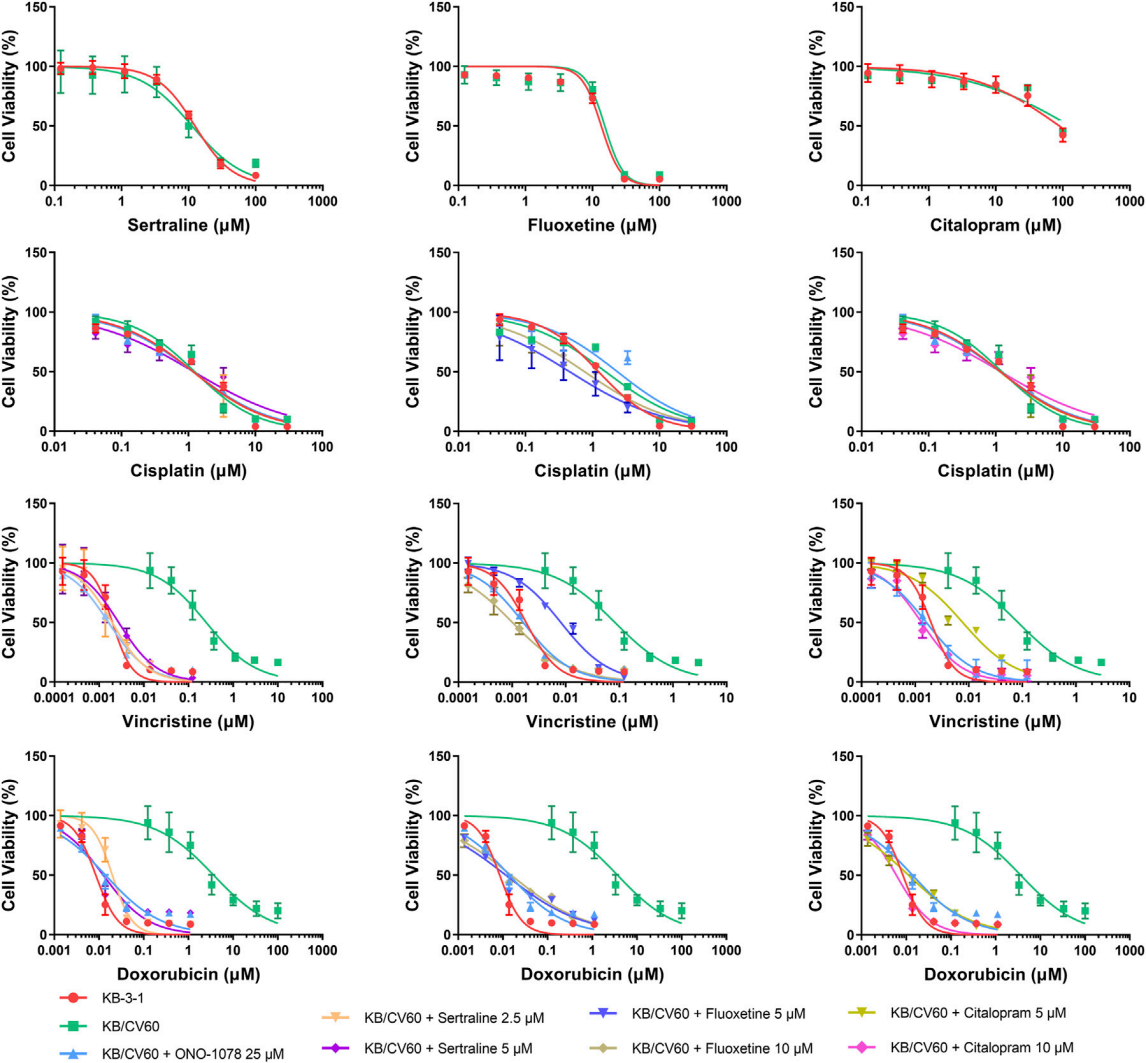
MTT assay showing the ability of sertraline, fluoxetine, and citalopram to reverse MDR mediated by MRP1 over-expressed in KB/CV60 cells. Mean ± SD, n = 3.

**FIGURE 6:**
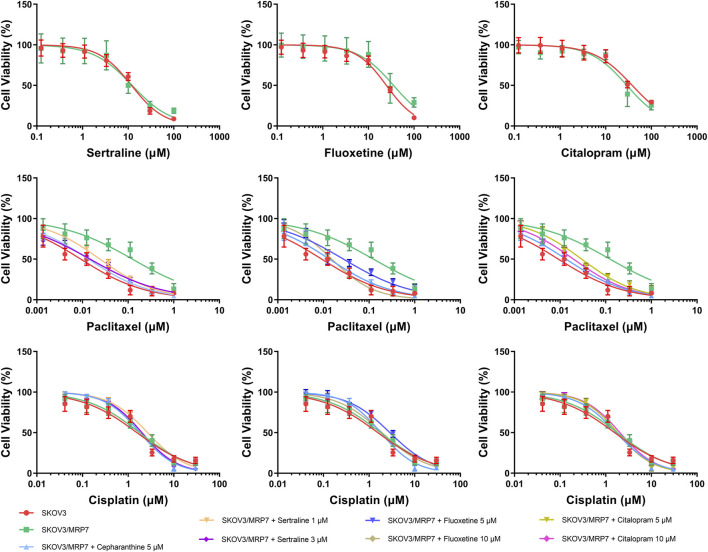
MTT assay showing the ability of sertraline, fluoxetine, and citalopram to reverse MDR mediated by MRP7 over-expressed in SKOV3/MRP7 cells. Mean ± SD, n = 3.

An MTT assay was performed to examine the cell viability of sertraline, fluoxetine, and citalopram on the pair of KB-3-1 and KB/CV60 cells (Figure 5) and the pair of SKOV3 and SKOV3/MRP7 cells (Figure 6), respectively. After that, the concentrations of sertraline, fluoxetine, and citalopram with no toxic (lower than IC_20_) were chosen to test the potential reversal effect by the MTT assay of substrate drugs with or without an inhibitor. Table 4 shows that the decreased cytotoxicity of the substrate drugs, vincristine, and doxorubicin, can be enhanced to a similar level with the known MRP1 inhibitor ONO-1078 and even similar to the cytotoxicity in the parental KB-3-1 cells. At the same time, the decreased cytotoxicity of the substrate drug, paclitaxel, can be enhanced to a similar level with the known MRP7 inhibitor cepharanthine and even similar to the cytotoxicity in the parental SKOV3 cells (Table 5). In addition, all three drugs and ONO-1078 and cepharanthine did not alter the cytotoxicity of the non-substrate drug, cisplatin, in either the drug-resistant cells or the parental cells.

**TABLE 4:** Effect of sertraline, fluoxetine, and citalopram on reversing MRP1-mediated MDR in KB/CV60 cells. Resistance Fold was calculated by dividing the IC_50_ value of a substrate drug into drug-resistant KB/CV60 cells in the presence or absence of an inhibitor (sertraline, fluoxetine, citalopram, or ONO-1078) by the IC_50_ value of parental KB-3-1 cells without an inhibitor.

Treatment	IC_50_ (Mean ± SD, nM) [Resistance fold]	
	KB-3-1	KB/CV60
Vincristine	1.98 ± 0.27 [1.00]	249.6 ± 27.73 [126.06]
VCR + Sertraline (2.5 μM)	1.96 ± 0.33 [0.99]	1.74 ± 0.18 [0.88]
VCR + Sertraline (5 μM)	1.74 ± 0.26 [0.88]	1.97 ± 0.27 [0.99]
VCR + Fluoxetine (5 μM)	1.83 ± 0.22 [0.92]	12.82 ± 2.13 [6.47]
VCR + Fluoxetine (10 μM)	1.61 ± 0.18 [0.81]	1.56 ± 0.17 [0.79]
VCR + Citalopram (5 μM)	1.98 ± 0.27 [1.00]	8.34 ± 1.61 [4.21]
VCR + Citalopram (10 μM)	1.77 ± 0.14 [0.89]	1.51 ± 0.26 [0.76]
VCR + ONO-1078 (20 μM)	1.62 ± 0.23 [0.82]	1.77 ± 0.21 [0.89]
Doxorubicin	8.48 ± 1.30 [1.00]	672.3 ± 88.50 [79.28]
DOX + Sertraline (2.5 μM)	9.18 ± 1.77 [1.08]	19.85 ± 2.08 [2.34]
DOX + Sertraline (5 μM)	8.19 ± 0.94 [0.97]	12.50 ± 1.53 [1.47]
DOX + Fluoxetine (5 μM)	8.67 ± 1.09 [1.02]	16.75 ± 1.63 [1.98]
DOX + Fluoxetine (10 μM)	9.28 ± 1.68 [1.09]	12.57 ± 1.56 [1.48]
DOX + Citalopram (5 μM)	8.40 ± 0.92 [0.99]	9.33 ± 1.04 [1.10]
DOX + Citalopram (10 μM)	6.05 ± 0.97 [0.71]	6.07 ± 0.84 [0.72]
DOX + ONO-1078 (20 μM)	8.98 ± 0.74 [1.06]	7.25 ± 0.92 [0.85]
Cisplatin	1778 ± 253.4 [1.00]	1839 ± 230.4 [1.03]
Cisplatin + Sertraline (2.5 μM)	1672 ± 249.4 [0.94]	1755 ± 198.5 [0.99]
Cisplatin + Sertraline (5 μM)	1852 ± 242.9 [1.04]	1794 ± 172.9 [1.01]
Cisplatin + Fluoxetine (5 μM)	1868 ± 162.6 [1.05]	1842 ± 191.0 [1.04]
Cisplatin + Fluoxetine (10 μM)	1725 ± 192.2 [0.97]	1827 ± 238.4 [1.03]
Cisplatin + Citalopram (5 μM)	1795 ± 177.5 [1.01]	1688 ± 152.7 [0.95]
Cisplatin + Citalopram (10 μM)	1758 ± 181.5 [0.99]	1776 ± 215.7 [1.00]
Cisplatin + ONO-1078 (20 μM)	1833 ± 107.7 [1.03]	1769 ± 229.0 [0.99]

Values in the table are represented as means ± SD determined from at least three independent experiments performed in triplicate.

**TABLE 5:** Effect of sertraline, fluoxetine, and citalopram on reversing MRP7-mediated MDR in SKOV3/MRP7 cells. Resistance Fold was calculated by dividing the IC_50_ value of a substrate drug into drug-resistant SKOV3/MRP7 cells in the presence or absence of an inhibitor (sertraline, fluoxetine, citalopram, or cepharanthine) by the IC_50_ value of parental SKOV3 cells without an inhibitor.

Treatment	IC_50_ (Mean ± SD, nM) [Resistance fold]	
	SKOV3	SKOV3/MRP7
Paclitaxel	9.21 ± 0.998 [1.00]	124.6 ± 23.8 [13.5]
PTX + Sertraline (1 μM)	8.55 ± 0.824 [0.93]	19.5 ± 2.16 [2.12]
PTX + Sertraline (3 μM)	8.57 ± 0.853 [0.93]	13.6 ± 2.13 [1.45]
PTX + Fluoxetine (5 μM)	9.53 ± 0.987 [1.03]	27.6 ± 2.52 [3.00]
PTX + Fluoxetine (10 μM)	9.20 ± 0.962 [1.00]	16.7 ± 1.85 [1.81]
PTX + Citalopram (5 μM)	9.28 ± 0.984 [1.01]	17.8 ± 1.82 [1.94]
PTX + Citalopram (10 μM)	9.96 ± 1.08 [1.08]	15.2 ± 1.71 [1.65]
PTX + Cepharanthine (5 μM)	8.68 ± 0.952 [0.94]	10.6 ± 1.17 [1.15]
Cisplatin	1752 ± 178 [1.00]	1787 ± 175 [1.02]
Cisplatin + Sertraline (1 μM)	1656 ± 183 [0.95]	1793 ± 193 [1.02]
Cisplatin + Sertraline (3 μM)	1784 ± 175 [1.02]	1786 ± 181 [1.02]
Cisplatin + Fluoxetine (5 μM)	1812 ± 185 [1.03]	1816 ± 188 [1.04]
Cisplatin + Fluoxetine (10 μM)	1766 ± 175 [1.01]	1789 ± 196 [1.02]
Cisplatin + Citalopram (5 μM)	1872 ± 185 [1.07]	1857 ± 199 [1.06]
Cisplatin + Citalopram (10 μM)	1794 ± 198 [1.02]	1713 ± 184 [0.98]
Cisplatin + Cepharanthine (5 μM)	1846 ± 187 [1.05]	1732 ± 183 [0.99]

Values in the table are represented as means ± SD determined from at least three independent experiments performed in triplicate.

## 4 Discussion

ABC transporters, which are responsible for the efflux of drugs from cells, are considered promising targets for drug discovery. By developing inhibitors of these transporters, multidrug resistance (MDR) can potentially be overcome and drug accumulation in cells can be improved. This study aims to investigate the repurposing of FDA-approved medications, specifically drugs from the SSRI family that are commonly used as antidepressants, to address drug resistance caused by ABCC1/MRP1 and ABCC10/MRP7, when exposed to chemotherapy. The research approach combines *in silico* predictions with *in vitro* validations to achieve synergy in the results obtained.

To examine the molecular characteristics of the human MRP1 and MRP7 in an atomic-scale resolution, it is necessary to analyze their structures. We previously generated these structures by homology modeling and subsequent molecular dynamics simulations (Amram et al., 2014; Bin Kanner et al., 2021; Wang et al., 2021d). However, our models were based on the cryo-EM bovine MRP1 that lacked stretches of tenths of residues and TMD0 or on the SAV1866 bacterial transporter. Therefore, in our current study, we opted to use the AlphaFold-generated MRP1 and MRP7 human structures as they include the missing gaps from the bovine MRP1 structure and TMD0. AlphaFold is currently a prominent source of protein structures that have not been experimentally determined. The AlphaFold models of the human MRP1 and MRP7 are consistent with the topological distinctive architecture of long eukaryotic ABCC transporters, as elaborated in Figure 1, left panel. By conducting comprehensive quality assessment analyses, we have determined that the AlphaFold-generated models can be considered robust and sound structures that are suitable for further in-depth structural and functional applications. Noteworthy, MRP1 and MRP7 possess structural differences, that may account for their distinct substrate specificities and transport activities.

Before performing docking experiments, we utilized the PSN-ENM method to gain a comprehensive understanding of the distinct characteristics and similarities between MRP1 and MRP7 and to reveal the unique binding modes of these two proteins. Specifically, we focused on the TMDs domains, which are of high interest due to their role in substrate binding and translocation. Two network analyses were conducted: hubs and meta-path. Hubs analysis identified highly connected nodes within the protein network, which may correspond to residues or regions of the protein that are involved in multiple interactions. Hubs that are within the TMDs have a high potential to be involved in ligand binding and to serve as druggable sites. Meta-path analysis involves examining the influence of different types of paths between nodes, providing insights into functional relationships between residues and how they contribute to protein function and stability.

We analyzed the networks of MRP1 and MRP7 to identify similarities and differences between the two proteins. The common hubs and links can be considered points of convergence in the communication pathways between residues within MRP1 and MRP7, revealing important similarities in the functional relationships between residues within these two proteins. We located 46 shared hubs in TMD1 and TMD2 that correspond to interacting residues, including K332/K292, M338/L298, P343/P303, H382/H342, and Y384/Y344 in MRP1/MRP7, respectively. These hubs were later found in the docking experiments to bind the SSRI drugs, which provided additional confirmation of our docking results. Surprisingly, in the meta-path analysis, we found only four links and eight nodes shared by both meta-paths. Six out of eight of the mutual regular nodes (not hubs) within the meta-paths were also found later in the docking experiments to bind the SSRI drugs: P343/P303, H382/N342, I591/M546, E1204/Q1156, G1207/G1159, and T1241/T1195 in MRP1/MRP7, respectively. Although the mutual node F583/F538 is not part of the binding site, it is specifically interesting since we previously reported that the point mutation F583A results in a long-range allosteric impact, that propagates across the membrane, rendering the protein inactive (Weigl et al., 2018; Bin Kanner et al., 2021). The unique hubs and links in the networks of MRP1 and MRP7 suggested differences in their binding specificity, and analyzing these unique links could identify key residues or interactions specific to each protein. For instance, the protein-specific hubs include H335, F385, and Q450 for MRP1, and Q1156 for MRP7. Our docking analyses further confirmed that these residues were involved in ligand binding only in one protein.

Molecular docking was performed using Maestro by Schrödinger suite to investigate the binding modes of sertraline, fluoxetine, and citalopram to MRP1 and MRP7 (Figure 3). It is imperative to visually evaluate the docking poses and consider other factors, such as the strength and type of chemical interactions between the protein and ligand, the conformational stability of the complex, and the biological relevance of the docking pose. In the case of MRP1, the binding sites of the drugs comprised mainly of hydrophobic and polar residues, with some charged residues also involved in binding (Figure 3, left panel). This aligns with previous studies, which divided the binding pocket into two sections: a positively charged region (the P-pocket) and a primarily hydrophobic area (the H-pocket) (Johnson and Chen, 2017; He et al., 2021). While the binding sites of MRP7 and MRP1 share hydrophobic and polar residues, MRP7’s binding site contains five glycine residues, whereas MRP1’s binding site has only one (as shown in Figure 3, right panel). Glycine residues are thought to play a role in enhancing flexibility at enzyme active sites (Yan and Sun, 1997). The presence of glycine residues in MRP7’s binding site confers localized flexibility, presumably facilitating the binding of a variety of ligands and compensating for the protein’s relative overall rigidity in comparison to MRP1. The different orientations of the drugs bound to each protein, as depicted in Figure 3 (left panel vs right panel), corroborate our observation of distinct levels of flexibility between the two proteins.

We identified several key amino acid residues in MRP1 and MRP7 that are involved in binding all three drugs, as well as some residues that were specific to certain drugs, as elaborated in Table 1. To evaluate the biological relevance of the docking pose, it is important to determine whether the docking pose is consistent with known biological data and if it is likely to be biologically active. In the ligand-bound bovine cryo-EM structures of MRP1 (Johnson and Chen, 2017; Pietz et al., 2023), important residues involved both in LTC4 and macrocyclic peptide binding were identified, including K332 and H335 (forming hydrogen bonds) and L381, F385, and F594 (forming hydrophobic contacts). It has been demonstrated that the transport of many substrates is significantly reduced or completely lost when these residues are mutated (Haimeur et al., 2002; Campbell et al., 2004; Haimeur et al., 2004; Situ et al., 2004). Similarly, the mutation of W1246 led to a loss of drug resistance and specifically affected the transport of organic anions (Ito et al., 2001). The outward-facing bovine cryo-EM structure study revealed that local structural changes in the substrate-binding site are necessary for substrate recognition and release outside the cell. Several residues, including F594 and Y1242 (corresponding to T1242 in humans), reposition their side chains to coordinate LTC4 during the transition from the apo to substrate-bound conformation. F583 was also found to be essential in exposing the translocation pathway to the extracellular space. In a study on the functional sites in human MRP1, P343, H382, Q450, F583, and R593 in TMD1, and S1235 in TMD2 were found to have relatively high ΔΔG values, highlighting their importance as key residues (He et al., 2021). The mutation N590A was reported to decrease the affinity of hMRP1 for LTC4 and substantially reduced the binding of ATP to NBD1 (Zhang et al., 2004). For MRP7, we previously predicted the potential binding pocket of inward-facing MRP7 based on the homology modeling of the bovine MRP1 cryo-EM structure (Wang et al., 2021d). In that study, we suggested that A542, R545, M546, and D1152 were residues involved in ligand binding. Furthermore, using docking experiments we presented the interaction of Paclitaxel and Methotrexate with the following residues: G299, A334, G337, A338, Q341, R545, M546, D1152, Q1156, L1192, S1193, T1195, G1196.

Halogenated compounds, including both synthetic and naturally occurring substances, have become increasingly popular in recent years for their diverse range of biological activities. The incorporation of halogen groups in the design of therapeutic agents has led to the development of innovative drugs with improved pharmacological properties (Benedetto Tiz et al., 2022). Accordingly, we found that sertraline and citalopram interact with various residues in the binding pocket of the MRPs through halogen bonds (as elaborated in the results section). This finding is consistent with previous studies showing that halogen-containing molecules can interact with ABC transporters. For instance, halogenated chalcones exhibited high binding affinity to P-gp (Bois et al., 1998), halogenated methylpurines were used as a substrate for MRP1 (Okamura et al., 2009; Zoufal et al., 2019), and halogenated derivatives of flavone-based compounds showed MDR-reversing capacity in MRP1 expressing cells (Mavel et al., 2006). Our results suggest that the presence of halogen groups in the chemical structure of SSRIs may contribute to their ability to inhibit MRPs. This insight provides valuable information on the mechanism of action of halogenated drugs and their potential therapeutic applications.

To determine the binding energy between the proteins and each of the drugs, three energy calculation methods were used: glide energy, MM-GBSA using Prime, and MM-GBSA using fastDRH. It is important to note that the values provided in Table 3 are based on the approximations used in the Glide software of Maestro and the MM-GBSA formalism. Therefore, these values should be considered estimates that indicate qualitative trends rather than precise quantitative values. The results revealed several important trends. First, the differences between the drugs were relatively small in each method, suggesting that all the ligands bind with similar affinity to the proteins. Second, the binding energy of the drugs to the two proteins differed, indicating that MRP1 and MRP7 may have slightly different binding characteristics for the three drugs. Furthermore, in two of the three methods, citalopram had the best score for MRP1, and fluoxetine had the best score for MRP7. Apart from MRP7-fluoxetine, the MRP1-ligand complexes consistently received higher rankings than the MRP7 complexes in most of the evaluations. This observation could be attributed to the innate flexibility of the proteins, as indicated by our NMA analysis. Specifically, MRP1 demonstrated greater structural flexibility, featuring numerous highly mobile regions that could facilitate its ability to bind and transport a diverse range of substrates. Conversely, MRP7 exhibited a more rigid structure, with fewer flexible regions, which could limit its substrate specificity and transport efficiency. Finally, the results suggest that different energy calculation methods may yield comparable outcomes, indicating the reliability of the findings.

In the *in vitro* study, the cytotoxicity of sertraline, fluoxetine, and citalopram were first assessed in MRP1- or MRP7-overexpressing cell lines, and the non-toxic concentrations were used in the following reversal studies. The results indicated that the drug resistance of the KB/CV60 and SKOV3/MRP7 were significantly resensitized after the co-incubation with sertraline, fluoxetine, and citalopram. However, the sensitivity of the parental cells were not affected. Meanwhile, no difference was suggested between the IC_50_ of cisplatin, which is not a substrate for either MRP1 or MRP7. Different chemotherapies were used in order to examine the potential inhibitory effect of the chemosensitizers. These drugs were chosen because they represent different modes of action: paclitaxel and vincristine are microtubule-targeting agents, cisplatin forms covalent bonds with DNA and by that causes intra-strand and inter-strand cross-links that interfere with DNA replication and transcription, and doxorubicin intercalates into DNA and inhibiting topoisomerase II. Taken together, the *in vitro* study results demonstrated the potential capability of sertraline, fluoxetine, and citalopram on reversing MRP1- and MRP7-related MDR.

The development of a new anti-cancer drug is a time-consuming process, taking up to 15 years and costing around $650 million (Prasad and Mailankody, 2017; Antoszczak et al., 2020). Therefore, it is reasonable to explore alternative strategies, such as drug repurposing. Our study examined the potential of repurposing drugs that have already received regulatory approval for one disease to treat alternative therapeutic applications for different medical conditions. Specifically, we explored the potential of SSRIs, which are primarily used to inhibit serotonin reuptake, as inhibitors of ABC transporters. Since cancer patients may already be taking SSRIs for depression and anxiety, we investigated whether these drugs could also act as chemosensitizers for MRP1 and MRP7, extending their potential benefits. Our findings were supported by clinical studies, including evidence that sertraline can enhance the effects of vincristine and doxorubicin (Amit et al., 2009) and inhibit the growth of colon cancer cells in colorectal cancer-xenografted mice (Gil-Ad et al., 2008). Interestingly, not only MRP1 and MPR7 but also P-gp serve as a target for chemosensitizers such as sertraline, fluoxetine (O’Brien et al., 2012), and citalopram (Uhr and Grauer, 2003; Uhr et al., 2008).

Our study makes a valuable contribution to the existing body of knowledge in this area of research from several aspects. First and foremost, it demonstrates that FDA-approved drugs can be repurposed for treating cancer, specifically overcoming chemotherapy resistance, saving valuable time and money. Second, our results suggest that the incorporation of halogen groups in the design of novel therapeutic agents could be a promising strategy to improve their pharmacological properties and aid in the development of more potent and selective MRPs inhibitors. In addition, to the best of our knowledge, this is the first study to use the predicted structures of the human MRP1 and MRP7 generated by AlphaFold. This is particularly significant for the human MRP7, one of the least studied members of the ABCC subfamily, which currently has no high-resolution structure available.

## Data Availability

The original contributions presented in the study are included in the article/Supplementary Material, further inquiries can be directed to the corresponding authors.
