# Supplementary figures and images for: Cytotoxicity and reversal effect of sertraline, fluoxetine, and citalopram on MRP1- and MRP7-mediated MDR
Source: Front Pharmacol. 2023 Nov 2;14:1290255. doi: 10.3389/fphar.2023.1290255 (PMC10651738; doi:10.3389/fphar.2023.1290255)

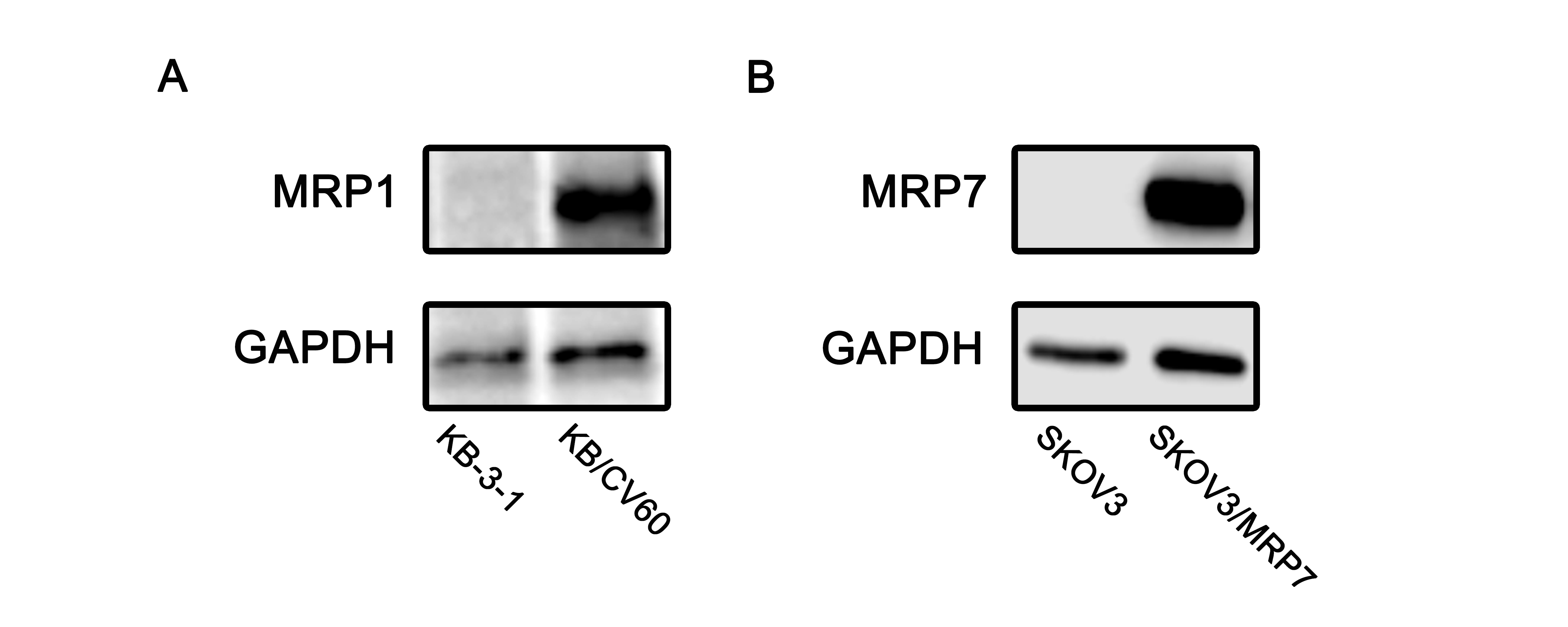

Supplement: Supplementary file 1 [file Image1.tif]
